# Multiple fractures after low-energy trauma in an immunosuppressed lung transplant patient with severe osteoporotic bone texture—a challenge for traumatology and osteology

**DOI:** 10.1093/jscr/rjae597

**Published:** 2024-09-26

**Authors:** Julian Ramin Andresen, Martin Direder, Harald K Widhalm

**Affiliations:** Department of Orthopedics and Trauma Surgery, Medical University of Vienna, Vienna, Austria; Department of Orthopedics and Trauma Surgery, Medical University of Vienna, Vienna, Austria; Department of Orthopedics and Trauma Surgery, Medical University of Vienna, Vienna, Austria

**Keywords:** lung transplantation, multiple fractures, low-energy trauma, immunosuppressed patient, osteoporotic bone texture, specific antiosteoporotic medication

## Abstract

Patients before and after lung transplantation often have osteoporosis with existing and recent symptomatic insufficiency fractures, which reduce the quality of life and increase general morbidity and mortality. Due to the reduced bone quality with a significantly increased fracture risk, even low-energy trauma results in the risk of acquiring multiple and complex fractures. The rarefied bone substance can be very challenging for subsequent osteosynthetic treatment. Antiosteoporotic medication is always necessary, and osteoanabolic therapy should be discussed in such cases. In the following, we report about the successful osteosynthetic treatment of an immunosuppressed patient with multiple fractures after a fall from low height. To support fracture healing with antiosteoporotic drug therapy, the patient was switched from antiresorptive to osteoanabolic medication, which resulted in complete fracture consolidation over the course of 6 months. There were also no new insufficiency fractures during this period; however, no improvement in bone density was achieved.

## Introduction

Patients with a status postlung transplantation are highly likely to develop osteopathy with significantly reduced bone mineral density and rarefaction of the bone structure due to long-term immunosuppressive medication, which consecutively leads to an increased fracture risk [1–5]. The occurrence of insufficiency or trauma-related fractures worsens the quality of life and increases morbidity and mortality [4].

## Case report

We report about an immunosuppressed, lung transplanted patient with multiple fractures after a fall from a height of 1 meter.

## Medical history

The patient had a history of manifest osteoporosis with previous rib and vertebral fractures and a *T*-score of −5 in the DEXA measurement (Lunar iDXA®, GE HealthCare, Germany) of the lumbar spine due to several years of cortisone therapy. In addition, there was a vitamin D deficiency of < 10 ng/ml. This was followed by antiosteoporotic medication with vitamin D, calcium, and the bisphosphonate alendronate (Fosamax®), which was subsequently switched to denosumab (Prolia 60®) due to the onset of renal insufficiency. Six months after the start of antiresorptive therapy, femoral head necrosis developed on both sides and hip replacement was indicated. Eighteen months before the accident, a lung transplant was performed due to chronic, thromboembolic pulmonary hypertension in the end stage. Furthermore, arterial hypertension and chronic ischemic heart disease were known.

## Newly acquired fractures

**Figure 1:**
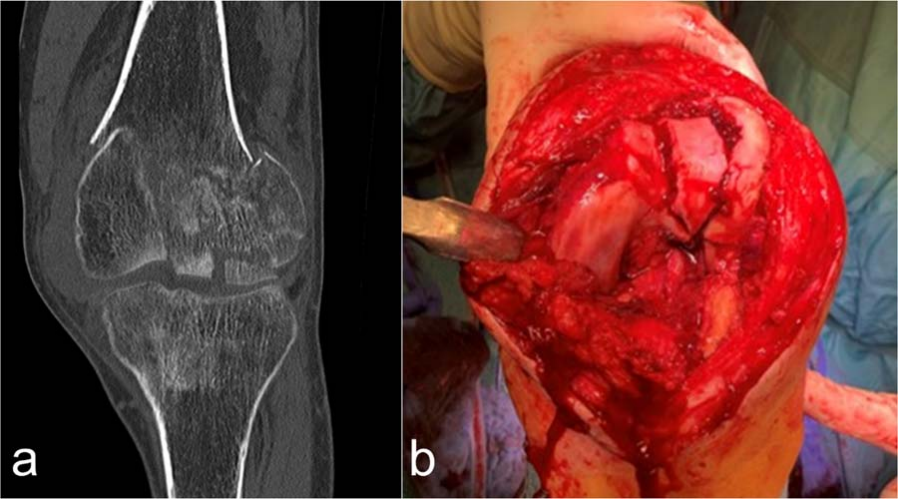
Case presentation of the knee region. (a) The coronal CT segment shows a transcondylar multifragment fracture of the femoral bone. (b) The intraoperative situs shows the comminuted zone of the condylar articular surface.

In the 48-year-old man with a body mass index of 18.4 kg/m^2^, after initial treatment in the emergency room, conventional imaging and CT examination revealed a supra-intracondylar multiple fragment fracture of the femoral bone (33C3. 2) (Fig. 1a), a subcapital dislocated humerus fracture (11C3.1), a radius fracture with involvement of the caput and collum radii (2R1C3 and Mason stages II and III) and a distal tibia with Maisonneuve fracture (44C2.2 and Weber C). The fracture classification was based on the AO-Trauma International Board & the Orthopedic Trauma Association [6].

## Surgical treatment

**Figure 2:**
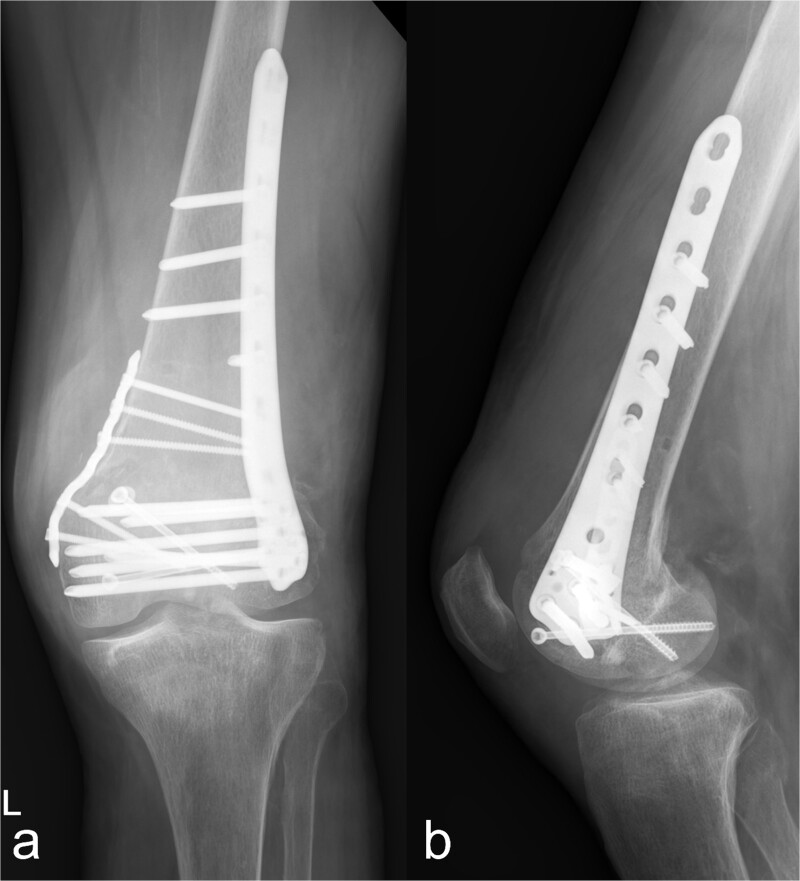
Treatment patterns of supra-intracondylar multifragment fracture (33C3.2). (a) The anterior-posterior and (b) lateral radiographs show a correct position of the osteosynthesis material with an nearly anatomical fracture position.

Osteosynthetic treatment via an open approach to the knee joint presented the multifragmentary femoral fracture radiating into the joint with an osseous avulsion of the lateral collateral ligament and posterolateral insertion of the anterior cruciate ligament (Fig. 1b). After reduction of the fracture fragments with temporary fixation using K-wires, an LCP 6-hole plate (Synthes®) was positioned medially and a 7-hole LISS plate (Synthes®) laterally and anchored with multiple screws (Fig. 2a and b). The osseous avulsion of the anterior cruciate ligament was fixed with a 3.5-mm SharkScrew®, the avulsion of the lateral collateral ligament with two FibreWire Loops®.

**Figure 3:**
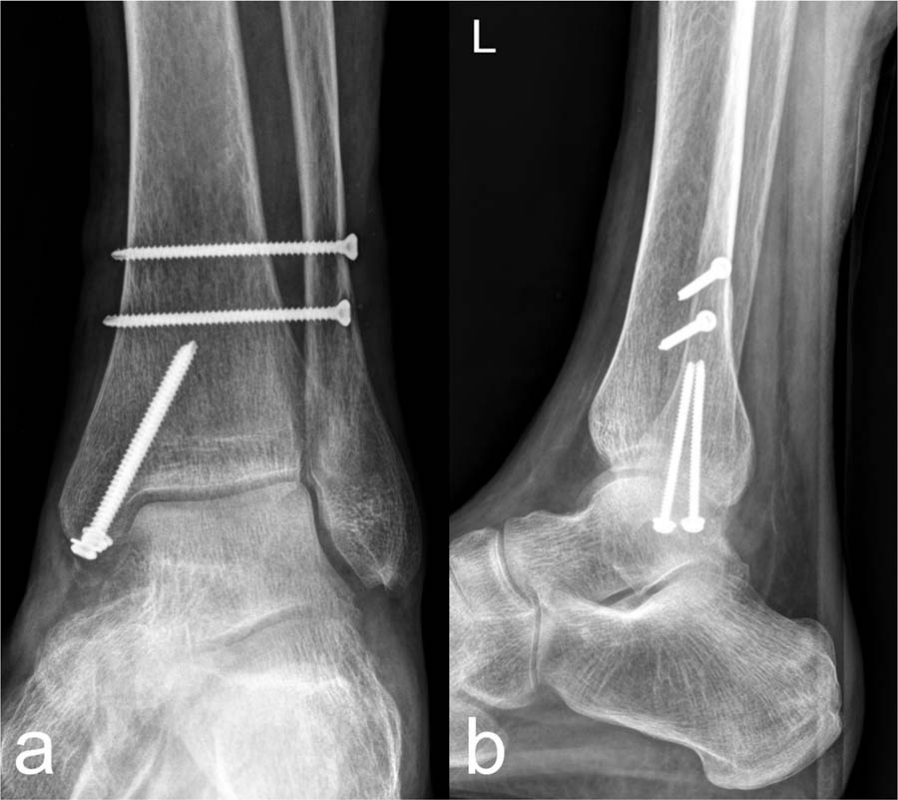
Treatment patterns of the distal tibia and Maisonneuve fracture (44C2.2 and Weber C). As a secondary finding, there is an osteopenic bone texture of the imaged skeletal area. (a) The anterior-posterior image shows an anatomical reposition of the medial malleolus, which was fixed with two lag screws. Two syndesmosis screws are located slightly further cranially. (b) The lateral image shows a continuous fragment adaptation of the medial malleolus/Vollkmann's triangle. The screws inserted at the level of the medial malleolus and proximal to the syndemosis tibiofibularis show a regular position.

An osseous fragment of the medial malleolus with Volkmann’s triangle was continuously adapted with two lag screws. In addition, the syndesmosis tibiofibularis was stabilized with two positioning screws (Fig. 3a and b).

**Figure 4:**
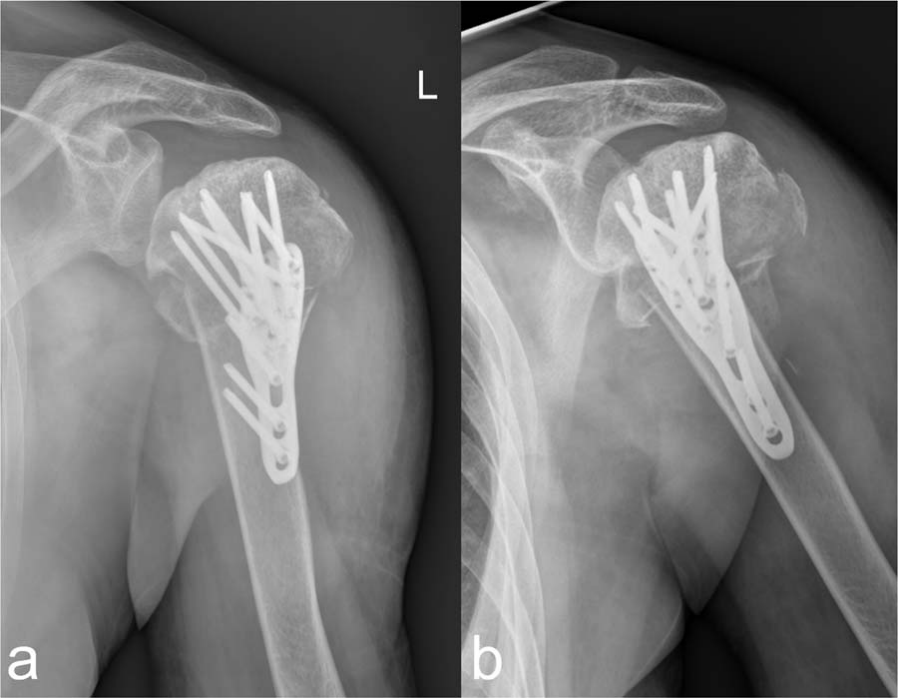
Treatment patterns of the subcapital humerus fracture (11C3.1). (a, b) The radiographs in two planes show extensive coverage of the fracture fragments with adequate stabilization of the multiple fragment fracture of the caput humeri, which was treated osteosynthetically using a PHILOS® plate and multiple screws.

**Figure 5:**
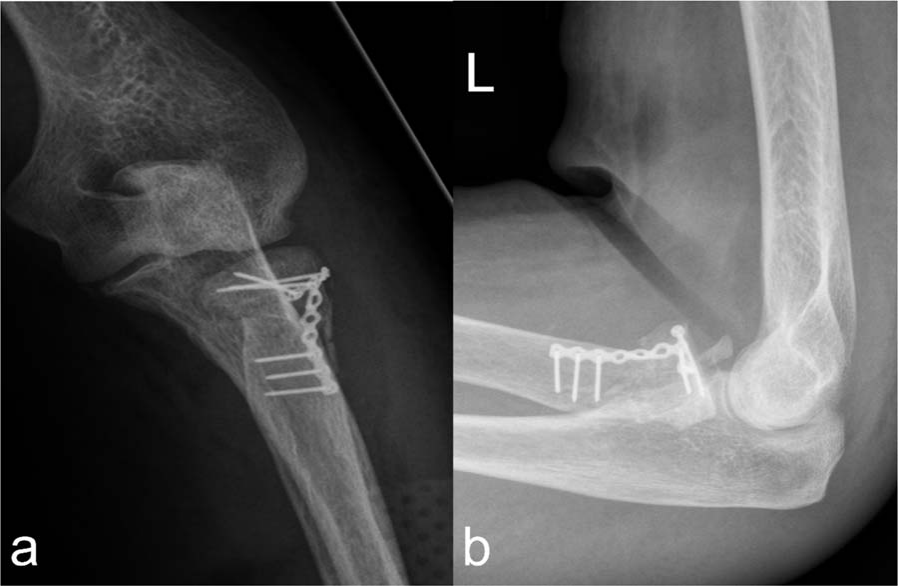
Treatment patterns of fractures of the caput and collum radii (2R1C3 and Mason stages II and III). (a, b) X-ray in two planes shows extensive coverage of the fracture fragments of the caput radii when the collum radii is straightened by the osteosynthesis material.

Using a 3-hole LCP plate (PHILOS-Synthes®) for the humerus (Fig. 4a and b) and a locking T-plate with two additional screws (Synthes®) for the caput and collum radii (Fig. 5a and b), adequate reduction and mostly axial alignment of the fractures were achieved.

The distal os femoris/supra-intracondylar multifragment fracture (33C3.2) and the distal tibia with Maisonneuve fracture (44C2.2 and Weber C) were treated first, followed four days later in a second session by the subcapital humerus fracture (11C3.1) and fracture of the caput/collum radii (2R1C3 and Mason stages II and III).

## Bone metabolism

Due to the manifest osteoporosis and to improve fracture healing [7], the antiresorptive medication was switched from denosumab (Prolia 60®) to the osteoanabolic substance teriparatide (Forsteo®) in accordance with the guidelines [8]. Over the course of 6 months, consolidation of the osteosynthetically treated fractures developed. There were no new insufficiency fractures during that period. However, the follow-up DEXA measurement showed no improvement in bone density in the lumbar spine with a *T*-score < −5.

## Discussion

In the fractures described, the osteopenic bone texture with rarefied spongiosa, thinning of the cortical bone and dislocated fragments present an intraoperative challenge in achieving a good reduction in an axially correct position and hardware fixation. In order to achieve the best possible primary stability in the acute situation and to minimize the occurrence of secondary fractures, locking plates, screws with different threads, intramedullary nails, wire cerclage, total endoprostheses, and additional PMMA cement augmentation are used [9–14]. In our patient, this was achieved by means of plate osteosynthesis and additional screws. In the complex multifragment fracture of the distal os femoris, lateral and medial plate insertion was performed to achieve sufficient stability (Fig. 2a and b). This minimizes the development of pseudarthrosis, while lateral and medial plate osteosynthesis does not have a negative influence on vascularity [15, 16]. However, even in nonorgan transplanted patients after optimal osteosynthesis of a distal femur fracture, a second surgery is necessary in every eighth patient [17]. In the case of multiple localizations with more complex fracture courses, several operations and follow-up procedures may be necessary. This necessitates extensive postoperative care, especially in organ transplant patients. Chiou *et al.* [18] report a 62.5 % mortality rate over the course of 1076 days in lung transplant patients after treatment of a fracture of the lower extremity.

## Conclusion

Due to the additional presence of manifest osteoporosis, osteoanabolic medication should be chosen to improve fracture healing and minimize fracture risk, as long as there are no known contraindications [7, 19]. For our patient, taking into account the relevant safety aspects and approval modalities, teriparatide (Forsteo®) was the only possible treatment option [20].

## Data Availability

Data generated during and/or analyzed during the current study are available from the corresponding author on reasonable request.
